# Long-term memory for a learned behaviour in a wild bird

**DOI:** 10.1098/rsbl.2019.0912

**Published:** 2020-02-12

**Authors:** Rachael C. Shaw, Annette Harvey

**Affiliations:** School of Biological Sciences, Victoria University of Wellington, Wellington 6012, New Zealand

**Keywords:** long-term memory, learning, avian cognition, conservation

## Abstract

Long-term memory is a crucial adaptation for long-lived species. However, there have been few tests of the long-term retention of learned behaviours in free living, wild animals. Here, we demonstrate that the North Island robin (*Petroica longipes*; hereafter toutouwai) can recall a learned foraging behaviour for close to 2 years, with no intervening reinforcement. Birds that had been trained to peck open lids to retrieve a concealed food reward spontaneously solved a lid opening task between 10 and 22 months since they had last encountered the lid opening apparatus. By contrast, naive individuals could not solve the task. This long-term retention of a learned skill with no reinforcement, spanning over a quarter of the median age for wild toutouwai in our population, suggests that this threatened species may be an ideal candidate for conservation management strategies aimed at teaching individuals about novel threats and resources.

## 1. Introduction

Across the animal kingdom, long-term memory underpins behaviours that are crucial for survival. From elephant matriarchs remembering the location of water sources during droughts [1], to caching corvid species retrieving thousands of stored seeds [2], multiple adaptive behaviours and behavioural traditions require the ability to recall information over periods of months or years. However, despite the adaptive importance of long-term memory, direct empirical tests for the long-term retention of novel information or behaviours remain scarce. There is some evidence that animals can recall novel foraging skills that they have either learned socially, or by trial and error. In captive studies of goats and lions, animals that have discovered how to open a novel puzzle box containing food remain more efficient at solving the task several months later, compared to naive individuals [3,4]. Similarly, an arbitrary foraging tradition (the preference for opening a either a blue or red door to retrieve food) was found to persist for nine months in wild great tits [5].

Understanding the limits and duration of memories for novel information and behaviour is important. Long-term memory is not only crucial for the development and persistence of behavioural traditions [5], but can also inform whether and how animals may adapt to a rapidly changing world. Human altered environments create evolutionarily novel conditions in which the ability to remember recurring novel foraging opportunities and avoid recurring novel threats may aid individual survival [6,7]. As yet, however, few studies have specifically examined whether wild animals retain information about a learned, novel foraging opportunity or threat over an extended time period.

We tested the long-term retention of a learned foraging behaviour in a relatively long-lived passerine. The North Island robin (*Petroica longipes*; here we use their Māori name, toutouwai) is a small insectivorous bird endemic to the North Island of New Zealand. Individuals can live upwards of 10 years in areas with no introduced mammalian predators [8]. Toutouwai can be readily trained using behavioural shaping procedures in the wild [9,10] and are highly territorial [11]. Moreover, as a caching species, it is likely that aspects of toutouwai memory performance are under direct selection [8]. These characteristics make it an ideal species for testing long-term memory in the wild. We trained birds to solve a novel foraging task and assessed whether they retained the learned skill for at least one full annual breeding cycle, with no intervening reinforcement of the behaviour or contact with the apparatus.

## 2. Method and materials

### (a) Study site and subjects

**Figure 1.**
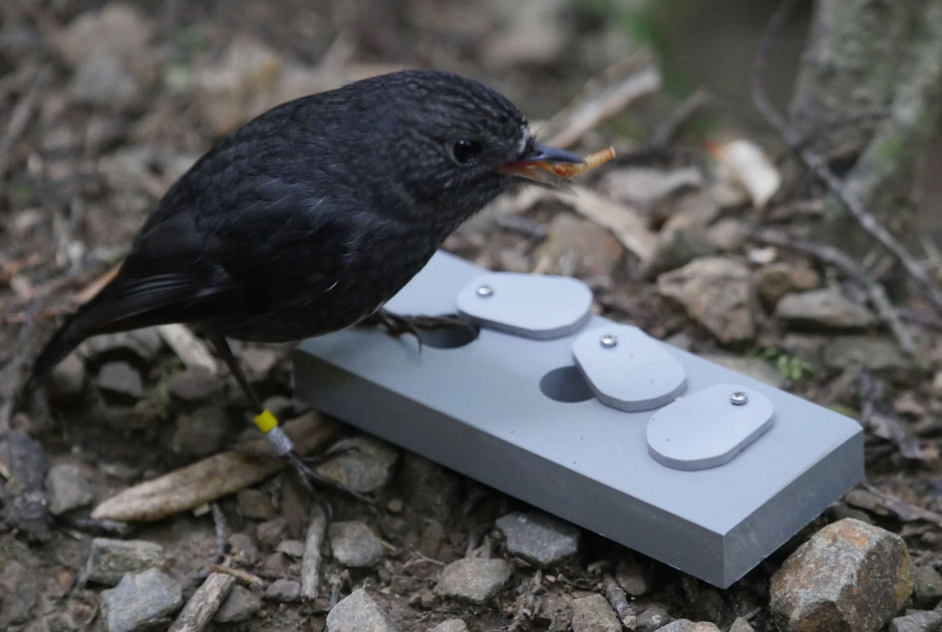
A toutouwai eats a mealworm after opening a lid. See electronic supplementary material video for a clip of an experienced and a naive bird interacting with the apparatus.

The toutouwai study population is located within a 25-hectare area at Zealandia Wildlife Sanctuary in Wellington, New Zealand. Since 2014 the population has been monitored and all territory holders and their offspring have been banded with a unique combination of three leg band colours for individual identification. In 2015 and 2016, we successfully trained all resident, territory holding adults (*N* = 64) to peck open a swivel lid to retrieve a mealworm from a hidden compartment (the apparatus is shown in figure 1, the lid opening shaping procedure is described in detail in [10]). For this study, we tested whether 32 of the trained birds (12 female, 20 male) retained this learned behaviour in 2018, a median of 468 days since they had last opened lids (range 313–938 days). We also tested 17 naive, untrained birds (5 female, 12 male) as controls (*N* = 7 in 2018, *N* = 10 in 2019). These control subjects were not trained in 2015–2016, either because they were resident outside the study area during this time, or because they were hatched after 2016. However, eight of the naive control birds were familiar with the apparatus, having previously eaten mealworms offered adjacent to it during an unpublished social learning experiment (median 946 days prior, range 337–1025) and one 2018 control subject had previously learned a different lid opening task in 2014 [9]. We tested birds from 22 January 2018 to 24 April 2018 and from 9 October 2019 to 21 October 2019, between 10.00 and 16.00. At the time of testing, birds were a median of 4 years old (range 0.5–11 years).

### (b) Experimental protocol

We gave all birds three trials to test their ability to peck open lids and retrieve a concealed food reward. The trials took place in a single testing session on a bird's territory, at least 5 m from a territory boundary. One bird in the experienced group and one bird in the naive group participated in only one trial, as they flew off after their first trial and did not return within 45 min. Prior to testing, birds hopped on an electronic scale to retrieve a mealworm larva (*Tenebrio molitor*). This ensured that toutouwai were food motivated; all birds took the worm. We began testing as soon as the bird had retrieved the mealworm from the scale. For each test, we placed the apparatus on the ground with the two outer wells baited with a freshly killed mealworm and lids fully closed. The apparatus was baited out of view and the middle well was left open and empty to serve as a visual reminder of the hidden compartments. A Go-Pro camera was placed on the ground nearby, *ca* 40 cm away from the apparatus and trials were also filmed on an iPad.

A trial started once the experimenter had stepped 2 m back from the apparatus. If a bird did not approach the apparatus within 2 min, to ensure it did not entirely lose interest and leave the area, a single mealworm was thrown on the ground within 10 cm of the apparatus. All of the birds that were given this mealworm retrieved and ate it. Trials ended when a bird had opened both lids, or after 5 min had elapsed. We allowed up to 2 min between trials to reset the apparatus and record the previous trial outcome. A bird solved a trial when it pecked open both lids to retrieve the mealworms within 5 min. From the videos, we counted the number of pecks the bird made on the apparatus. If applicable, we also measured the total time taken to retrieve both worms, as well as the number of pecks and time taken to retrieve the first worm only. To ensure consistency in timing measurements between individuals, we began measuring the time it took to open lids from the first time a bird approached within two body lengths of the apparatus. To exclude neophobia toward the apparatus as an explanation for the lack of pecking behaviour in naive birds, in 2019 we added an additional step for control birds in which we opened both lids at the end of the third and final trial, so that the mealworms were visible inside the compartments. All 10 control subjects tested in 2019 took the mealworms within seconds of seeing them.

### (c) Statistical analysis

We analysed data in R using linear and generalized linear (mixed) models (lme4 package [12]) with multi-model inference (MuMIn package [13]). For all birds, we modelled the total number of apparatus pecks in each trial using a generalized linear mixed model, with a Poisson error structure. We specified a global model containing experience (naive = 0, trained = 1), subject age, sex and trial order as fixed factors, with subject ID as a random factor. For the solvers only, we used a linear mixed model for the time (log transformed) that it took to open the first lid. We excluded one male that had participated in only the first trial; doing so improved model fit and did not change the overall pattern of results. The global model included the number of days elapsed since the previous lid opening experience (retention interval), subject age, sex and trial order as fixed factors, with subject ID included as a random factor. Finally, for the solvers only we used a Poisson GLM to examine how retention interval, sex and age influenced the number of pecks required to open the first lid in the first trial.

For each global model described above, we created a model set by running all possible combinations of the predictors. In electronic supplementary material, table S1, we report the five best-fitting models in each set, together with their Akaike information criterion corrected for small samples sizes (AICc), the change in AICc relative to the best model in the set (ΔAICc) and the Akaike weight (AICw), which gives the conditional probability of the model [14]. We then averaged across all possible models in a set to obtain averaged model parameter estimates and 95% confidence intervals (95% CI) [14]; these are reported in electronic supplementary material, table S2.

## 3. Results

**Figure 2.**
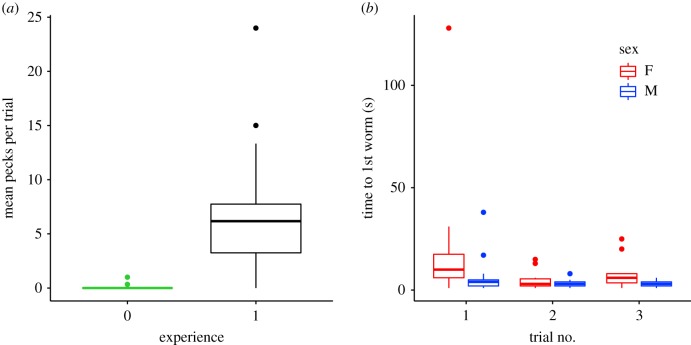
(*a*) The mean number of total apparatus pecks per trial for naive (green; group ‘0’) and experienced (black; group ‘1’) birds. (*b*) The time that individuals took to open the first lid during each trial (females in red, males in blue). In both plots, outliers are shown as points, the whiskers show range (excluding outliers), the boxes depict interquartile range and the bold centre line shows the median value.

Of the 32 experienced toutouwai, 30 birds spontaneously solved the task, opening both lids on their first trial. By contrast, none of the 17 naive birds solved the task (electronic supplementary material, table S3). Experienced birds were also far more likely to peck the apparatus than naive birds (mean *β* ± s.e. = 4.270 ± 0.519, 95% CI = 3.245 to 5.295; figure 2*a*). Examining solvers' behaviour revealed that males were slightly faster to retrieve the first worm compared to females (mean *β* ± s.e. = −0.650 ± 0.236, 95% CI = −1.119 to −0.180; figure 2*b*), but that the number of days that had elapsed since a bird last solved the task (i.e. the retention interval) did not affect the time it took to open the first lid (see electronic supplementary material, table S2). There was a tendency for toutouwai to take longer to retrieve the first worm in their first trial (mean *β* ± s.e. = −0.243 ± 0.101, 95% CI = −0.443 to −0.043; figure 2*b*). However, this effect was largely driven by a single female who took 128 s to approach in her first trial. Finally, the number of pecks required to open the first lid in the first trial was not influenced by retention interval, age or sex (see electronic supplementary material, tables S1 and S2).

## 4. Discussion

The overwhelming majority of toutouwai retained the ability to open lids for over a year since they had last encountered the task. This result did not arise because these birds were more likely to attempt to interact with the apparatus and rediscover how to open the lids by trial and error. The experienced birds’ pecking behaviour was spontaneous and targeted. On average they pecked only twice to open the first lid, right from their very first trial. By contrast, naive birds almost never pecked the apparatus, even if they had previously been fed beside it, or participated in other, similar experiments [9]. This was not simply because naive birds avoided the apparatus; all readily ate a mealworm placed near it and naive birds tested in 2019 also ate directly from the apparatus. Taken together, these results strongly suggest that toutouwai are able to retain novel, learned foraging behaviour for almost 2 years in the wild. As other non-caching passerines have been shown to recall a socially learned foraging preference for several months in the wild [5], it seems likely that accurate, long-term recall for learned foraging innovations is an ability shared by many passerine species.

For solvers, the time since they had last encountered and solved the task ranged from 10 to 22 months. Yet there was no effect of retention interval on either the time or number of pecks that experienced birds required to open the first lid. The only two experienced birds that failed to open any lids fell at either end of the retention interval range (321 days and 938 days, respectively). However, the female that did not recall the task after 938 days had previously been excluded from a spatial learning experiment requiring lid opening 2 years prior (austral winter 2016 [8]), as she had already lost the lid opening ability by this time (after a 405-day retention interval). Our results strongly suggest that retention interval duration is not correlated with forgetting, or with an increasing reliance on trial and error learning to re-learn specific aspects of the task. Moreover, as the majority of toutouwai displayed highly accurate task recall regardless of the retention interval length, it is possible that birds may have been capable of remembering the task over a longer period than we were able to test in the current study.

Birds in our study recalled a novel foraging behaviour over an interval that covered more than a quarter of their median age when tested. This long-term retention of a learned behaviour opens up the exciting possibility of harnessing learning and memory for toutouwai conservation management [15]. Across New Zealand, toutouwai, along with many other species, have been introduced into ‘mainland island sanctuaries' [16], which are designed to eliminate the threat posed by non-native mammalian predators. However, as mammalian-predator naive birds disperse and attempt to establish in surrounding areas, only those individuals that can avoid predation [17] and exploit novel resources [15] are likely to survive. The memory ability demonstrated in our experiment suggests that toutouwai are likely to be excellent candidates for conservation interventions that aim to teach naive individuals about such threats before they disperse.

## Supplementary Material

Supplementary Information

## Supplementary Material

R code

## Supplementary Material

Dataset

## Supplementary Material

Video

## Data accessibility

The data and code supporting this article have been uploaded as part of the electronic supplementary material.
