# Supplementary material for: Long-term memory for a learned behaviour in a wild bird
Source: Biol Lett. 2020 Feb 12;16(2):20190912. doi: 10.1098/rsbl.2019.0912 (PMC7058958; doi:10.1098/rsbl.2019.0912)
Supplement: Supplementary Information [file rsbl20190912supp1.pdf]

Shaw RC, Harvey A. Long term memory for a learned behaviour in a wild bird. ***Biology Letters***

Table S1: Support for the top five models for the analyses of total pecking behaviour across all birds (N = 49) and two measures of solver behaviour (*N* = 30; one solver was excluded from the 'time (log) to open 1st lid' analysis, as he participated in only the first trial). The intercept only model was the 10th ranked model for the total pecks analyses and we have included it for reference. AICc is the small sample Akaike Information criteria, ΔAICc is the change in AICc relative to the top model, AICw (Akaike weight) is the conditional probability of the model.

**Response**	**Model**	**AICc**	**ΔAICc**	**AIC*w***
total pecks during all trials (all birds, *N* = 49)	1. experience + trial no. + age	625.39	0	0.19
	2. experience + trial no.	625.52	0.12	0.18
	3. experience + age	625.94	0.55	0.14
	4. experience	626.08	0.68	0.13
	5. experience + trial no. + age + sex	626.53	1.14	0.11
	10. intercept only	701.81	76.42	0.00
time (log) to open 1st lid in all trials (solvers only, *N* = 29)	1. sex + trial no.	233.81	0	0.47
	2. sex	234.45	0.64	0.34
	3. trial no.	237.42	3.61	0.08
	4. intercept only	238.12	4.31	0.06
	5. age + sex + trial no.	239.89	6.08	0.02
no. of pecks to open 1st lid in trial 1 (solvers only, *N* = 30)	1. age	90.64	0	0.31
	2. intercept only	91.14	0.50	0.24
	3. age + retention interval	92.78	2.15	0.11
	4. age + sex	92.87	2.23	0.10
	5. retention interval	93.12	2.48	0.09

Table S2: Multi-model averaged parameters for the analyses of total pecking behaviour across all birds (N = 49) and of two measures of solver behaviour (N = 30; one solver was excluded from the 'time (log) to open 1st lid' analysis, as he participated in only the first trial). For each predictor we list the relative variable importance (RVI, calculated as the sum of AIC weights for all models containing the predictor), the mean regression coefficient (β), the SE and 95% CI for mean β.

Response	Predictor	RVI	Mean β	SE	95% CI		
total pecks during trial	*experience*	*1*	*4.270*	*0.519*	*3.245*	*to*	*5.295*
	trial no.	0.57	-0.081	0.050	-0.179	to	0.017
	age	0.52	-0.092	0.061	-0.212	to	0.028
	sex	0.36	0.264	0.266	-0.263	to	0.790
time (log) to open 1st lid	*sex*	*0.86*	*-0.650*	*0.236*	*-1.119*	*to*	*-0.180*
	trial no.	*0.58*	*-0.243*	*0.101*	*-0.443*	*to*	*-0.043*
	age	0.05	-0.017	0.060	-0.136	to	0.102
	retention interval	<0.01	<0.001	0.001	-0.002	to	0.002
no. of pecks to open 1st lid in trial 1	age	0.55	0.104	0.059	-0.017	to	0.225
	retention interval	0.26	<0.001	0.001	-0.002	to	0.003
	sex	0.25	0.150	0.289	-0.442	to	0.743

Shaw RC, Harvey A. Long term memory for a learned behaviour in a wild bird. ***Biology Letters***

Table S3: The average behaviour of the experienced (*N* = 32) and naïve (*N* = 17) toutouwai tested in the lid opening task in their first trial and across all trials. The group's mean value for each measure is shown, with the range given in brackets.

	**Experienced**	**Naïve**
$N_{solvers}$	30	0*
Mean days since last test for solvers	482 (313 – 683)	N/A
<u>Trial 1:</u>		
time to 1st worm (s)	11.9 (1 – 128)	N/A
no. pecks to 1st worm	1.9 (1 – 5)	N/A
total pecks	7.0 (0 – 61)	0
<u>All trials:</u>		
time to 1st worm (s)	6.9 (1 – 128)	224**
no. pecks to 1st worm	1.8 (1 – 5)	1**
total pecks	6.5 (0 – 61)	0.1 (0 – 3)

*Excludes a male that opened one lid with his foot 1 min into his final trial, and then pecked lid fully open 1 min later to retrieve the partially visible worm.

**These values include the single trial described above, as only one lid was fully opened by the naïve group in total.
